# An unusual cause of failure in Zenith Alpha Abdominal endograft

**DOI:** 10.1186/s40001-022-00656-5

**Published:** 2022-03-02

**Authors:** Raffaella N. Berchiolli, Michele Marconi, Irene Bargellini, Giulia Bertagna, Daniele Adami, Davide M. Mocellin, Roberto Cioni, Mauro Ferrari, Nicola Troisi

**Affiliations:** 1 Vascular Surgery Unit, Department of Translational Research and New Technologies in Medicine and Surgery, University of Pisa, Pisa, Italy; 2 Interventional Radiology Department, Azienda Ospedaliero Universitaria Pisana, Pisa, Italy; 3 Vascular Surgery Unit, Cardio Thoracic and Vascular Department, Pisa University Hospital, Via Paradisa 2, Pisa, Italy

**Keywords:** Abdominal aortic aneurysm, Endoleak, Device design, Endovascular aneurysm repair, Endovascular treatment/therapy

## Abstract

**Background:**

Graft disruption is an unusual complication of the endovascular abdominal aortic aneurysm repair (EVAR).

**Case presentation:**

A 71-year-old man underwent standard EVAR with Zenith Alpha Abdominal endograft. Follow-up examinations revealed an initial significant sac shrinkage. At 24 months, duplex ultrasound (DUS) scan and computed tomography showed increase of the sac diameter associated with complete disconnection of the suprarenal stent-graft from the main body without evidence of endoleak. A standard relining with a thoracic endograft was performed between the suprarenal stent and the main body of the previous graft. At 6 months DUS revealed sac shrinkage.

**Conclusions:**

This report demonstrates an uncommon cause of endograft failure with suprarenal stent disconnection from main body and highlights the need for continuous follow-up in patients undergoing EVAR.

## Background

Endovascular aneurysm repair (EVAR) has been widely recognized as an alternative to open surgery for the treatment of abdominal aortic aneurysm (AAA), because of its lower early morbidity and mortality rates, even if secondary interventions and late complications are more common [1].

Follow-up is a crucial point in all patients undergoing EVAR in order to avoid endograft-related complications and sac enlargement [2].

We here report an unusual case of disconnection of the bare suprarenal stent from the main body of a Zenith Alpha Abdominal endograft, which led to sac enlargement without evidence of endoleak.

## Case presentation

A 71-year-old man with a 63-mm infrarenal AAA underwent EVAR by using the Zenith Alpha Abdominal graft (Cook Medical; Bloomington, IN, USA) under general anesthesia.

AAA features covered the instructions for use (IFU) of the device including infrarenal aortic neck length (16 mm), maximum angulation (10°), neck diameter (23 mm), and absence of severe calcifications or thrombus. The diameters of the common iliac arteries were 16 mm on the right side and 14 mm on the left, respectively.

A main body ZIMB-26-84 was deployed via a bilateral femoral access with two iliac extensions (Zenith Flex ZSLE-16-56 ZT left side; ZSLE-20-56 ZT right side). No intraprocedural complication occurred. At completion angiography no endoleak was found after ballooning with Reliant balloon (Medtronic Vascular; Santa Rosa, CA, USA).

Hospital stay was uneventful. Patient was discharged on the 2nd postoperative day.

**Fig. 1:**
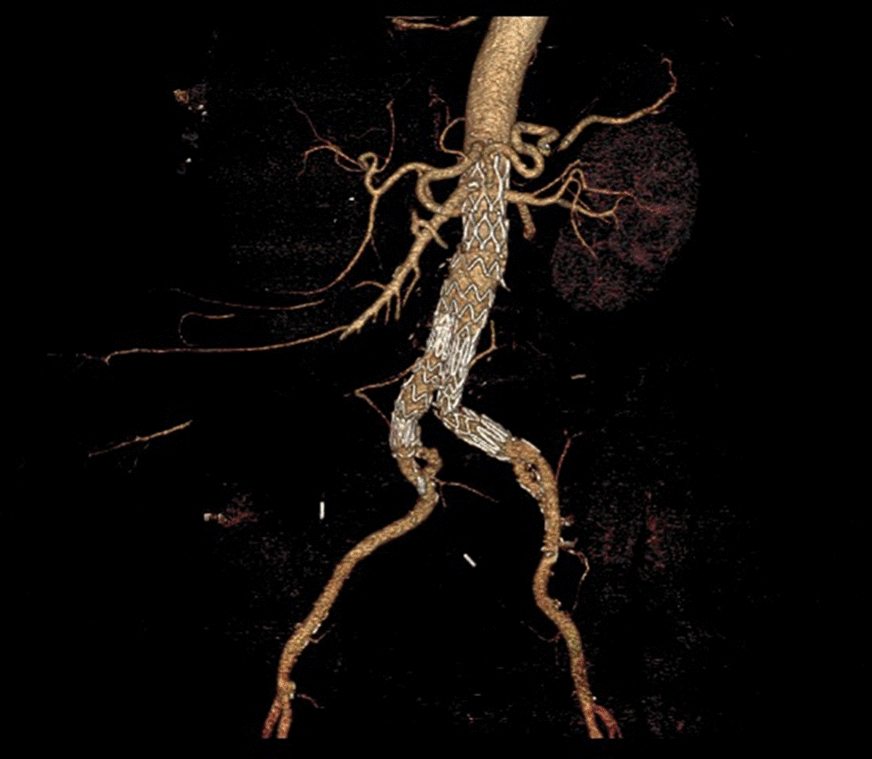
Postoperative CT-scan after initial EVAR

At 1 month computed tomography (CT) scan showed complete exclusion of the aneurysm without complications (Fig. 1).

Follow-up duplex ultrasound (US) at 6, 12, and 18 months detected a significant shrinkage of the aneurysmal sac from 63 to 38 mm without any signs of endoleak. However, at 24 months Duplex US revealed sac enlargement (54 mm; + 16 mm) without any evidence of endoleaks.

**Fig. 2:**
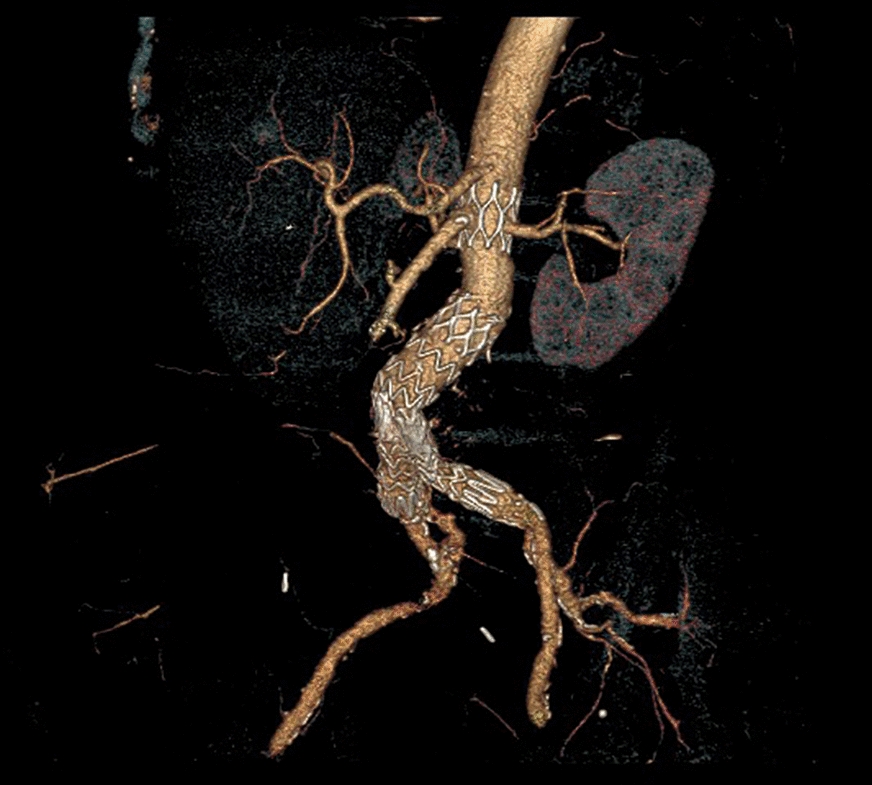
24-month CT-scan showing disconnection of the bare suprarenal stent from main body stent-graft

CT-scan revealed a complete disconnection of the suprarenal stent from the main body of the endograft. The latter was distally migrated with a concomitant severe kinking of the left iliac extension. No endoleak was detected (Fig. 2).

An endovascular relining procedure was planned and performed. The distance between the lowermost renal artery and the bifurcation of the previous bifurcated graft was 91 mm. A Zenith TX2 thoracic endograft (TBE-28-80-PF) was inserted and deployed via a redo surgical access on the right side. A Smart Control Stent 12 × 60 mm (Cordis, Cardinal Health; Dublin, Ireland) was placed into the left iliac extension via a percutaneous left femoral approach.

**Fig. 3:**
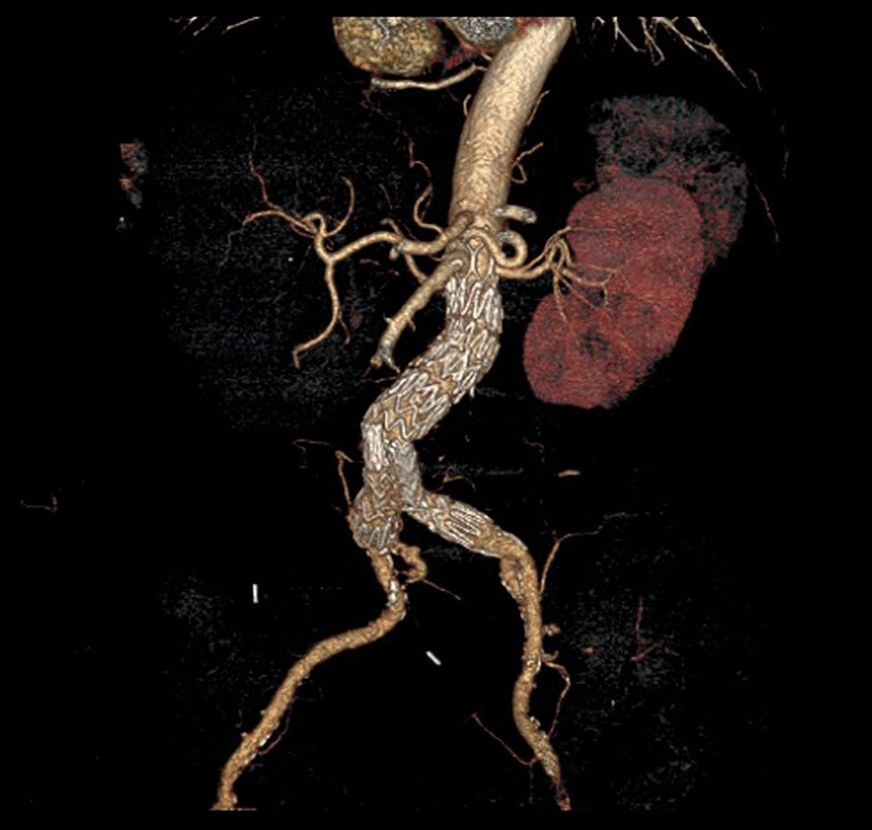
Postoperative CT-scan after redo EVAR

The postoperative CT-scan showed that the right renal artery was partially covered by the graft (Fig. 3). On the 2nd postoperative day a bare metal stent (RX Herculink Elite Renal Stent System 5 × 15 mm; Abbott CardioVascular, Plymouth, MN, USA) was successfully inserted and deployed via a left brachial access. After 6 months, at Duplex US the aneurysm sac was stable and both iliac extension were patent without any signs of stenosis.

The patient gave his consent to the publication of this report.

## Discussion

EVAR is a minimally invasive modality for AAA treatment associated with a reduced perioperative mortality rate compared to open repair. EVAR has become the preferred approach for the treatment of infrarenal AAA; however, long-term follow-up suggests that the survival benefits from EVAR are lost due to higher rate of reinterventions [1].

The causes of EVAR failure are multifactorial on the basis of anatomical features, not accurate preoperative planning, and device fatigue/failure.

About Zenith endograft, disconnections of the proximal uncovered stent have been reported in the literature with the first generation of this device prior to 2002 [3, 4]. Over the years, the stent-graft has been modified with a double-suture reinforcement to secure the uncovered stent to the graft. Since this structural modification has been introduced, stent disconnection between the modules has become very rare [5, 6].

In addition, no disconnections with the newest generation of Zenith devices (Zenith Alpha) have been previously reported. However, our case showed how this issue has not been definitively resolved with this new generation of endograft.

In our case, the connection between the suprarenal bare stent and the proximal part of the main body of the graft may represent an area of weakness. No endoleak was detected at imaging. Maybe the patient suffered from endotension. In fact, despite an initial AAA sac shrinkage, the disconnection occurred 2 years after EVAR, while previous reports have shown this complication to occur 3–8 years after treatment [5–8].

Careful re-review of preoperative planning confirmed that endograft sizing was made within the manufacturer’s IFU. Therefore, the anatomy gives no direct suggestions on endograft failure. In addition, the revision of the initial procedure did not show any problems about the post-implantation ballooning; the balloon was inflated in accordance with its own IFU.

This case has been reported to Cook Medical in order to improve current products and future designs, and an investigation has been initiated.

About technical issues, in our case a thoracic endograft was used because of the enough distance from the lowermost renal artery and the bifurcation of the previous main body. The length of the thoracic graft was 80 mm; it seemed to be the more appropriate device in order to guarantee the maximum overlap and the greatest columnar strength [9].

## Conclusion

Disconnection of the suprarenal stent in Zenith Alpha stent-graft is a rare complication not previously reported in literature. Deployment of a thoracic endograft to reline the modules was successful. This complication underlines the need for a continued life-long surveillance after EVAR, even with the use of newest generation devices.

## Data Availability

The datasets generated and analyzed during the current study are available from the corresponding author on reasonable request.
