# Correction to: An unusual cause of failure in Zenith Alpha Abdominal endograft

**DOI:** 10.1186/s40001-022-00680-5

**Published:** 2022-04-07

**Authors:** Rafaella N. Berchiolli, Michele Marconi, Irene Bargellini, Giulia Bertagna, Daniele Adami, Davide M. Mocellin, Roberto Cioni, Mauro Ferrari, Nicola Troisi

**Affiliations:** 1 Vascular Surgery Unit, Department of Translational Research and New Technologies in Medicine and Surgery, University of Pisa, Pisa, Italy; 2 Interventional Radiology Department, Azienda Ospedaliero Universitaria Pisana, Pisa, Italy; 3 Vascular Surgery Unit, Cardio Thoracic and Vascular Department, Pisa University Hospital, Via Paradisa 2, Pisa, Italy

## Correction to: European Journal of Medical Research (2022) 27:1–3 https://doi.org/10.1186/s40001-022-00656-5

In the original version of this article [1], the given and family names of the last author were swapped and incorrectly published as Troisi Nicola. The corrected author name should read as Nicola Troisi. The original article has been corrected.
